# New and recurrent *AAGAB* mutations in punctate palmoplantar keratoderma

**DOI:** 10.1111/bjd.12927

**Published:** 2014-08-07

**Authors:** E Pohler, M Huber, SE Boonen, M Zamiri, PA Gregersen, M Sommerlund, M Ramsing, D Hohl, WHI McLean, FJD Smith

**Affiliations:** 1 Centre for Dermatology and Genetic Medicine, Colleges of Life Sciences and Medicine, Dentistry and Nursing, University of DundeeDundee, U.K; 2 Department of Dermatology, University Hospital of Lausanne (CHUV)Lausanne, Switzerland; 3 Department of Clinical Genetics, Aarhus University HospitalAarhus, Denmark; 4 Department of Dermatology, University Hospital CrosshouseKilmarnock, U.K; 5 Alan Lyell Centre for Dermatology, Southern General HospitalGlasgow, U.K; 6 Department of Clinical Genetics, Aalborg University HospitalAalborg, Denmark; 7 Department of Dermatology and Venerology, Aarhus University HospitalAarhus, Denmark; 8 Department of Pathology, Aarhus University HospitalAarhus, Denmark

Dear Editor, Punctate palmoplantar keratoderma type I (PPPK1; also known as Buschke-Fischer-Brauer type; OMIM 148600) is an autosomal dominant disorder of keratinization, characterized by multiple hyperkeratotic lesions on the palms and soles that usually start in early adolescence but may also start later in life.^1^ Lesions increase in size and number with advancing age and may coalesce over pressure points to form larger plaques. Recently, two consecutive studies identified the causative gene for PPPK1 as *AAGAB*,^2^,^3^ located on chromosome 15q23, a locus to which the causal gene for PPPK1 was previously mapped.^4^ Subsequently, further mutations in *AAGAB* were described in families from several countries worldwide.^5^–^11^ In this study, we investigated five European families with a clinical diagnosis of PPPK1. This work was conducted in accordance with the principles of the Declaration of Helsinki. Blood was obtained following written informed consent and DNA extracted using standard protocols. Polymerase chain reaction amplification and Sanger sequencing to screen all exons and exon/intron boundaries of *AAGAB* was done as described previously.^3^

The 31-year-old proband from family 1, a four-generation family of Swiss origin (Fig.1a), presented with small, horny nails and hyperkeratosis of the palms and soles. Age of onset was 7 years. Discrete punctate lesions were observed along the lateral edge of the plantar surface, with more extensive hyperkeratosis over the heels (Fig.1b). Peeling skin was observed in the interdigital webspaces of the feet. The proband's father displayed extensive hyperkeratotic lesions on his palms (Fig.1c). We identified a previously unreported heterozygous nonsense mutation p.Gln88*; c.262C>T (Fig.1d,e) within the GTPase domain of the p34 protein (Fig.2e). This mutation is neither present in the Database of Single Nucleotide Polymorphisms (http://www.ncbi.nlm.nih.gov/SNP/) nor in the Exome Variant Server (http://evs.gs.washington.edu/EVS/). The resulting haploinsufficiency in this family is consistent with the increase in cell proliferation seen in PPPK lesions, as reported previously.^3^

**Fig 1:**
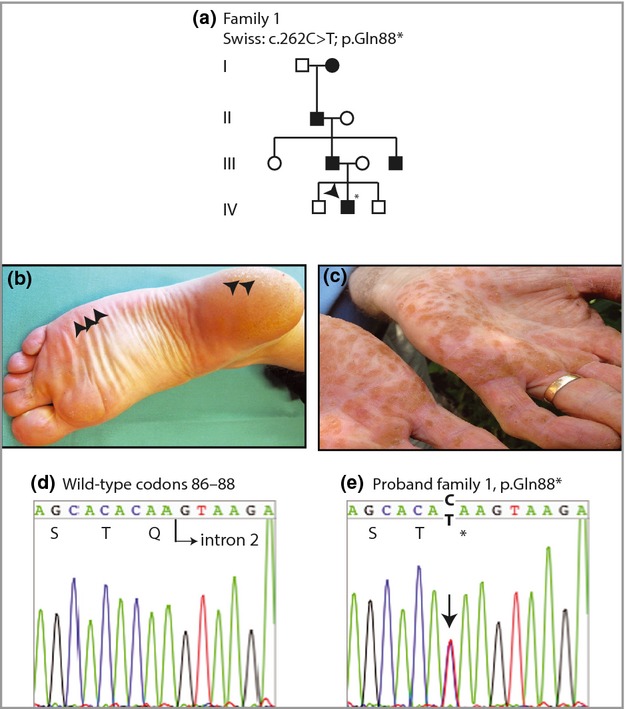
Pedigree, clinical images and mutation analysis of family 1. (a) Pedigree showing a four-generation history of punctate palmoplantar keratoderma. The arrowhead indicates the proband. (b) Punctate hyperkeratotic lesions (arrows) around the lateral edge of the foot and heel on the proband. (c) Palms of the father of the proband showing multiple hyperkeratotic lesions. (d) DNA sequence of codons 86–88 of *AAGAB* in an unaffected control sample. (e) The same region as in (d) from the proband of family 1. The arrow indicates the heterozygous C>T mutation at c.262 resulting in nonsense mutation p.Gln88*.

**Fig 2:**
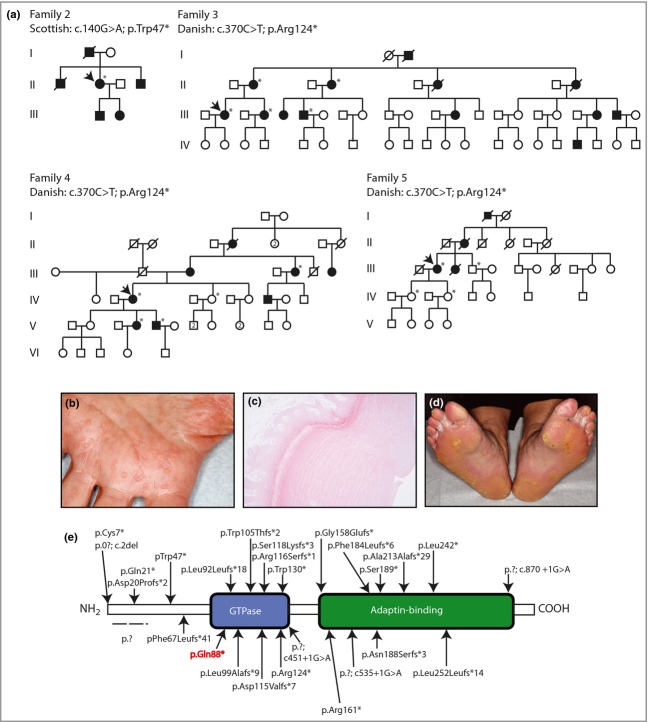
Pedigrees, clinical images and histology of punctate palmoplantar keratoderma type 1 families 2–5. (a) Pedigrees of the families with the relevant mutations indicated. Arrows denote the proband, and asterisks show individuals in whom *AAGAB* was sequenced. (b) The palm of the proband of family 3 showing the presence of multiple hyperkeratotic lesions. (c) Haematoxylin and eosin staining of a punch biopsy from palmar skin from the proband in family 3. This shows compact orthokeratosis with a defined central depression within the lesion. (d) The plantar surface of the index case in family 4. Hyperkeratotic lesions coalesce at pressure points. (e) Organization of the protein domains in p34 encoded by *AAGAB*, illustrating the location of all mutations reported to date. The p.Gln88* mutation highlighted in red is the novel mutation reported in this study.

In family 2, a 72-year-old Scottish woman was one of four family members over three generations with palmoplantar keratoderma (Fig.2a). She presented with a 1-year history of worsening hyperkeratosis affecting primarily her soles. Small hyperkeratotic lesions were seen on her fingers but not on her palms. Both her children were affected, with hyperkeratosis developing at a much younger age than in herself. Her two brothers were similarly affected, and her deceased father was reported to have been affected. A recurrent heterozygous nonsense mutation, p.Trp47*; c.140G>A,^3^,^5^ within the N-terminal domain of p34 (Fig.2e), was identified in the proband.

The other three families studied were apparently unrelated Danish kindreds. The 44-year-old proband of family 3 (Fig.2a) developed dry palmar skin in her childhood progressing to punctate keratosis on the palms (Fig.2b) and subsequently the soles. Haematoxylin and eosin staining of a biopsy from palmar skin (Fig.2c) showed compact orthokeratosis with pronounced hyperkeratosis. A central dermal depression characteristic for punctate palmoplantar keratoderma was observed. A recurrent heterozygous nonsense mutation Arg124*; c.370C>T^2^ was identified in the proband and four other affected members of this four-generation kindred (Fig.2a). PPPK1 was reported in four generations of family 4 (Fig.2a). The proband, a 53-year-old Danish female developed PPPK1 at 18–20 years of age. It gradually became worse, especially at pressure points on the feet (Fig.2d) where punctate lesions coalesced. The palms and volar sides of the fingers were affected to a lesser degree. The same p.Arg124* mutation as in family 3 was identified in the proband, her affected daughter, son and aunt but not in an unaffected sister (Fig.2a). In family 5, PPPK1 developed around the age of 20 years and progressed thereafter in the 68-year-old Danish proband. She presented with severe, very painful punctate palmoplantar keratoderma on the pressure points of her soles. Palms were also affected with multiple 3–5-mm punctate keratosis. Heterozygous nonsense mutation p.Arg124* was also identified in this individual. Three unaffected members did not carry the mutation (Fig.2a).

PPPK1, an autosomal dominant disorder characterized by multiple hyperkeratotic lesions on the palms and soles, can vary in severity from mild to severe, and can be painful and socially debilitating. Recently, mutations have been described in the *AAGAB* gene, which encodes the α- and γ-adaptin binding protein p34, and has been proposed to have a role in skin integrity.^2^ p34 has been functionally implicated in the intracellular transport of clathrin-coated vesicles,^3^ and may affect cell signalling via regulation of the expression of receptor tyrosine kinases, which are turned over by clathrin- and AP2-dependant mechanisms.^3^,^5^,^12^,^13^ Recently a mutation was reported in *AAGAB* in a Jewish family of Dutch origin with PPPK1 co-segregating with congenital dislocation of the hip.^10^ Whether this is a coincidence or if *AAGAB* has an as yet unknown role in skeletal development is unclear.

This study has revealed one novel, p.Gln88*, and two recurrent, p.Trp47* and p.Arg124*, mutations. This brings the total to 27 distinct loss-of-function mutations reported in *AAGAB* (Fig.2e), providing further evidence for this being the causative gene for PPPK1.

## References

[1] Emmert S, Küster W, Hennies HC (2003). 47 individuals in 14 families with the rare genodermatosis keratosis puncatata palmopantaris Buschke-Fischer-Brauer. Eur J Dermatol.

[2] Giehl KA, Eckstein GN, Pasternack SM (2012). Nonsense mutations in AAGAB cause punctate palmoplantar keratoderma type Buschke-Fischer-Brauer. Am J Hum Genet.

[3] Pohler E, Mamai O, Hirst J (2012). Haploinsufficiency for AAGAB causes clinically heterogeneous forms of punctate palmoplantar keratoderma. Nat Genet.

[4] Martinez-Mir A, Zlotogorski A, Londono D (2003). Identification of a locus for type 1 punctate palmoplantar keratoderma on chromosome 15q22–q24. J Med Genet.

[5] Pohler E, Zamiri M, Harkins CP (2013). Heterozygous mutations in AAGAB cause type 1 punctate palmoplantar keratoderma with evidence for increased growth factor signalling. J Invest Dermatol.

[6] Kiritsi D, Chmel N, Arnold AW (2013). Novel and recurrent AAGAB mutations: clinical variability and molecular consequences. J Invest Dermatol.

[7] Li M, Yang L, Shi H (2013). Loss of function mutation in AAGAB in Chinese families with punctate palmoplantar keratoderma. Br J Dermatol.

[8] Cui H, Gao M, Wang W (2013). Six mutations in AAGAB confirm its pathogenic role in Chinese punctate palmoplantar keratoderma patients. J Invest Dermatol.

[9] Li M, Dai X, Cheng R (2014). A novel 5-bp deletion mutation in AAGAB gene in a Chinese family with palmoplantar keratoderma. Acta Derm Venereol.

[10] Eytan O, Sarig O, Israeli S (2014). A novel splice-site mutation in the AAGAB gene segregates with hereditary punctate palmoplantar keratoderma and congenital dysplasia of the hip in a large family. Clin Exp Dermatol.

[11] Furniss M, Higgins CA, Martinez-Mir A (2014). Identification of distinct mutations in AAGAB in families with type I punctate palmoplantar keratoderma. J Invest Dermatol.

[12] Ceresa BP (2006). Regulation of EGFR endocytic trafficking by rab proteins. Histol Histopathol.

[13] Rappaport JZ, Simon SM (2009). Endocytic trafficking of activated EGFR is AP-2 dependent and occurs through preformed clathrin spots. J Cell Sci.

